# The Proapoptotic Effect of Traditional and Novel Nonsteroidal Anti-Inflammatory Drugs in Mammalian and Yeast Cells

**DOI:** 10.1155/2013/504230

**Published:** 2013-08-01

**Authors:** Gianluca Farrugia, Rena Balzan

**Affiliations:** Department of Physiology and Biochemistry, Faculty of Medicine and Surgery, University of Malta, Msida MSD 2080, Malta

## Abstract

Nonsteroidal anti-inflammatory drugs (NSAIDs) have long been used to treat pain, fever, and inflammation. However, mounting evidence shows that NSAIDs, such as aspirin, have very promising antineoplastic properties. The chemopreventive, antiproliferative behaviour of NSAIDs has been associated with both their inactivation of cyclooxygenases (COX) and their ability to induce apoptosis *via* pathways that are largely COX-independent. In this review, the various proapoptotic pathways induced by traditional and novel NSAIDs such as phospho-NSAIDs, hydrogen sulfide-releasing NSAIDs and nitric oxide-releasing NSAIDs in mammalian cell lines are discussed, as well as the proapoptotic effects of NSAIDs on budding yeast which retains the hallmarks of mammalian apoptosis. The significance of these mechanisms in terms of the role of NSAIDs in effective cancer prevention is considered.

## 1. Introduction

In recent years, there has been a growing interest in aspirin and other nonsteroidal anti-inflammatory drugs (NSAIDs) because of their promising antineoplastic properties. Epidemiological, clinical, and experimental evidence strongly indicates that NSAIDs exert a significant chemopreventive, antiproliferative effect on several types of cancer cells (see Table 1). Much of the research concerning the antineoplastic effects of NSAIDs has focused on the effect of aspirin in large bowel cancers, with comparatively fewer studies carried out on the chemopreventive effects of other NSAIDs [1]. In fact, aspirin stands as the most widely studied pharmacological agent for the chemoprevention of colorectal malignancies, with numerous clinical trials being carried out to examine its role in the prevention of adenomas, colorectal carcinomas, and inherited colorectal neoplasias such as the Lynch syndrome and familial adenomatous polyposis (FAP) [2]. Such focus on aspirin may be due, in large part, to the increasingly high prevalence and social impact of human colorectal cancer in recent years [2]. It is also due to the simple fact that long-term aspirin use is already widely practised among patients for the prevention of cardiovascular events such as thrombosis and neurovascular events such as stroke, thus providing a convenient opportunity for researchers to study its other long-term chemopreventive effects. In fact, major findings of aspirin's anticancer effects in humans are also derived from clinical trial data originally compiled for the study of its anti-thrombotic effects [3, 4]. Aspirin's affordability and ease of access, together with its relatively reduced side effects with respect to other traditional NSAIDs [5], have also helped to increase its appeal as a potential chemopreventive agent and target in anticancer studies. 

**Figure 1:**
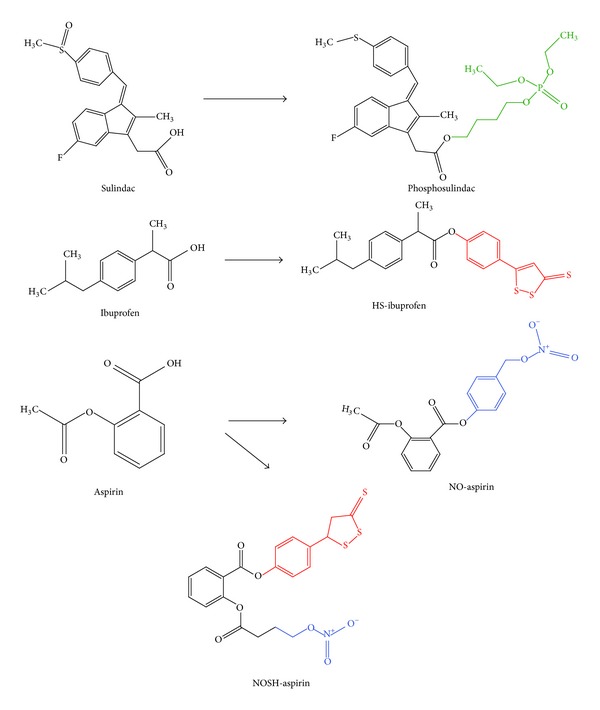
Chemical structures of modified NSAIDs and their traditional NSAID precursors. Phosphosulindac, which exemplifies phospho-NSAIDs, consists of the sulindac molecule linked at the –COOH site to a phosphate group *via* an aliphatic spacer molecule (both shown in green). In the HS-NSAID known as HS-ibuprofen, an aromatic spacer molecule links an H_2_S-releasing dithiolethione group (both shown in red) to the ibuprofen structure. Similarly, the NO-NSAID NO-aspirin is composed of an NO-releasing-NO_2_ group and an aromatic spacer molecule (both shown in blue) which is linked to the –COOH group of aspirin. Finally, the modified NSAID chimera known as NOSH-aspirin is characterized by the aspirin structure linked *via* two separate spacer molecules to both a H_2_S-releasing moiety (shown in red) and an NO-releasing moiety (shown in blue).

Nevertheless, a considerable number of investigations have shown that other NSAIDs including sulindac [6–8], indomethacin [9, 10], ibuprofen [11, 12], naproxen [13], and diclofenac [14–16] also exhibit significant antineoplastic behaviour in mammalian cancer cells. Additionally, recent studies have increasingly focused on the chemopreventive properties of a new NSAID class referred to as modified NSAIDs. These are essentially traditional NSAIDs which can either have nitric oxide- (NO-) donating moieties, hydrogen sulphide- (H_2_S-) donating moieties, or phosphate moieties covalently attached to the –COOH site *via* aromatic or aliphatic spacer molecules, as shown in Figure 1. The resulting modified NSAID classes, known as NO-donating NSAIDs (NO-NSAIDs), H_2_S-donating NSAIDs (HS-NSAIDs), and phospho-NSAIDs, respectively, have all been shown to be far less toxic than their NSAID precursors and several times more potent in terms of antineoplastic efficacy [17–22]. The exceedingly potent anti-neoplastic properties of novel NSAID chimeras, which are characterized by their possession of both NO- and H_2_S-donating moieties (see Figure 1), have also begun to attract significant attention [23, 24].

The mechanistic pathways which mediate the anti-neoplastic effects of traditional and modified NSAIDs are still not fully understood. It has been postulated that the antiproliferative effect of NSAIDs on malignant cells involves the inhibition of proinflammatory COX activity [25] and prostaglandin formation [26]. However, additional evidence shows that NSAIDs can induce apoptotic cell death in tumour cells [27] *via* pathways that are largely independent of COX [6, 28, 29]. The elucidation of apoptotic mechanisms underlying the chemopreventive effect of NSAIDs has long been the focus of intense research using a broad range of experimental models, including whole mammalian specimens, human cancer cell lines, and *Saccharomyces cerevisiae* cells. 

### 1.1. Yeast Cells as a Model for the Study of the Proapoptotic Effects of NSAIDs

Yeast cell species, such as *Saccharomyces cerevisiae*, are among the preferred and extensively used experimental models for the study of programmed cell death associated with ageing, disease, and cancers in living organisms. This is partly because yeast cells retain many core elements of mammalian cell processes such as apoptosis [30]. Additionally, these primitive eukaryotes have a number of important advantages over complex mammalian cell models. Yeast cells are relatively inexpensive and easy to handle, with a relatively brief lifespan that permits rapid generation of experimental results in a shorter span of time. Moreover, the yeast cell genome is very well characterised and relatively easy to manipulate, allowing for the ready availability of yeast mutant strains for experimental studies. Overall, these features collectively account for the successful use of budding yeast as a model organism for the study of molecular pathways underlying mammalian pathologies such as cancer.

 These same advantages of the yeast cell experimental model also account for its wide use in the study of proapoptotic pathways underlying the anti-neoplastic behaviour of antitumour drugs such as paclitaxel, bleomycin, valproate, and arsenic [31]. These compounds have been studied extensively in yeast cells in an effort to improve anticancer strategies in human patients. Likewise, *S. cerevisiae* budding yeast cells have also been used to study the growth inhibitory, proapoptotic effects of NSAIDs such as aspirin [32] and diclofenac [33]. 

The study of the proapoptotic effects of NSAIDs in yeast models is still a relatively new concept, with far fewer studies having been carried out in yeast with respect to mammalian cells. Regardless, evidence acquired thus far from yeast studies of NSAIDs such as aspirin has already yielded valuable insights into their proapoptotic behaviour, highlighting factors which play key roles in NSAID-induced death (such as reactive oxygen species (ROS) and mitochondrial dysfunction). In fact, compelling evidence has shown that *S. cerevisiae* cells constitute a powerful model for the screening and development of NSAIDs and other proapoptotic drugs designed for use in human cancer patients, overcoming the problem of cell specificity in the design of antitumour compounds [31], whilst also serving as an inexpensive model to initially test the effect of antitumour drugs before assaying them in more relevant mammalian systems. Therefore, yeast cells clearly serve an important role as a complementary experimental model to mammalian cells in the study and elucidation of NSAID-induced mechanisms of apoptosis. 

In this review, important biomolecular pathways triggered by traditional and novel NSAIDs which lead to the induction of apoptosis in mammalian cell lines and in *S. cerevisiae* yeast cells will be discussed. The significance of these proapoptotic mechanisms, in the context of the role NSAIDs may play in the design of more effective cancer prevention strategies, is also considered. 

## 2. NSAID-Induced COX Inhibition and Apoptotic Cell Death

The cyclooxygenase isoforms COX-1 and COX-2, both of which are key requirements for the synthesis of prostaglandins in mammalian cells, constitute the best defined pharmacological targets of NSAIDs such as aspirin [61, 62]. Thus, it has long been postulated that the chemopreventive effect of NSAIDs is mediated by their ability to inactivate COX-enzymes, causing inhibition of prostaglandin synthesis and consequent suppression of both tissue inflammation and cell proliferation, two conditions heavily associated with tumour formation [25]. In fact, both prostaglandins and COX enzymes, particularly COX-2, are implicated in tumorigenesis [1, 2, 63, 64] and have been observed in high quantities in several types of human tumours, including colorectal carcinomas [65–69]. Moreover, prostaglandins such as PGE_2_ are known to promote angiogenesis, alter cellular immunity, increase both proliferation and invasiveness of cells, and enhance cellular resistance to apoptosis [2, 68, 70]. 

It has been shown that deletion of receptors for PGE_2_ confers resistance to both polyp and cancer formation [71]. Similarly, disruption of COX-2 activity was found to reduce the incidence of polyp [72, 73] and aberrant crypt foci formation [74] in the intestines of rodent models. Inhibition of COX-2 has also been shown to be effective in preventing the formation of human colorectal adenomas [75, 76] and oesophageal squamous cell carcinoma [64]. Thus, inhibition of COX-2-dependent prostaglandin synthesis is thought to mediate, at least in part, the tumour-suppressive, antiproliferative effects of NSAIDs such as the suppression of angiogenesis and the induced lowering of resistance to apoptosis. In fact, inhibition of invasive tumour formation in NSAID-treated mouse models has been associated with decreased cellular levels of PGE_2_ [26].

The anti-neoplastic behaviour of NSAIDs is also associated with their ability to actively induce apoptosis in malignant cells. However, the means by which COX-2 inhibition could possibly mediate NSAID-induced cancer cell apoptosis has long been the subject of debate [77, 78]. As such, there is no clear evidence implicating direct involvement of prostaglandins in NSAID-induced apoptosis, which suggests that prostaglandins do not directly mediate NSAID-induced death [77–79]. In fact, studies have indicated that aspirin-induced apoptosis in mouse [80] and human [81] cancer cells can occur independently of prostaglandin inhibition. Similarly, Chan and coworkers [7] demonstrated that apoptosis induced by sulindac and indomethacin in mammalian HCT116 and SW480 colon cancer cell lines did not depend on prostaglandin depletion since supplementation of these same cells with prostaglandins failed to protect them from apoptosis. However, this same study did present evidence of COX-2-dependent apoptosis induced by these NSAIDs. The authors suggested that cellular accumulation of the prostaglandin precursor arachidonic acid, brought about by the NSAID-induced inhibition of COX-2, caused cancer cell apoptosis by stimulating sphingomyelinase-mediated synthesis of ceramide [7], which is a proapoptotic molecule [82]. Arachidonic acid accumulation in cancer cells also induces ROS accumulation [83], mitochondrial permeability transition, and cytochrome *c* release [84], all of which lead to apoptotic cell death.

## 3. COX-Independent NSAID-Induced Apoptotic Cell Death

The proapoptotic effects underlying the chemopreventive potential of NSAIDs cannot be accounted for by COX inhibition alone. Firstly, NSAIDs have been shown to inhibit proliferation and induce apoptosis in malignant cell lines which do not express either COX-1 or COX-2, as observed in cyclooxygenase-null mouse embryo fibroblasts [85] and human colon cancer cells [28, 81, 86]. Furthermore, NSAID compounds which cannot inactivate COX-2, such as sulindac sulfone, a metabolite of sulindac [87], have been shown to induce apoptosis of gastric tumour cells [88] and inhibit colon tumour formation in rodents [89, 90]. Additionally, NSAID concentrations required to induce apoptosis in cancer cells have often been found to be significantly higher than that required to inhibit COX-2, suggesting the presence of alternative cellular targets of NSAIDs [27, 37, 77, 91]. Indeed, numerous studies have shown that NSAID-induced apoptosis in mammalian tumour cells can be mediated by several largely COX-independent metabolic pathways, the most prominent of which are presented in the following discussion.

### 3.1. Activation of Caspases and Modulation of Bcl-2 Proteins

NSAIDs can mediate apoptosis by inducing the activation of caspases, a family of proapoptotic cysteine proteinase enzymes which typically exist as latent zymogens in cells. Activation of these proteins by proapoptotic signals initiates a caspase cascade whereby initiator caspases specifically activate other executioner-type caspases. The latter then degrade multiple cellular components so that cells begin to acquire the morphological and biochemical features of apoptosis [92]. Bellosillo and coworkers [37] were among the first to present evidence of NSAID-induced caspase activation. They showed that B-chronic lymphocytic leukemia (B-CLL) cells treated with high doses of aspirin underwent apoptosis characterised by DNA fragmentation and degradation of poly (ADP-ribose) polymerase (PARP), which is a caspase substrate. The apoptotic phenotype was inhibited by application of the pan-caspase inhibitor Z-VAD-FMK, thus confirming the involvement of caspases in aspirin-induced B-cell apoptosis. Similarly, Castaño and coworkers [34] affirmed caspase involvement in aspirin-induced apoptosis of HT-29 human colon adenocarcinoma cells. However, contrary to its effect in B cells, aspirin did not induce PARP degradation in HT-29 cells. This observation is probably one of the earliest to suggest that NSAID-induced activation of caspases can occur *via* different pathways. 

Indeed, it has since been shown that NSAIDs activate caspases both *via* the mitochondrial-mediated (intrinsic) pathway, which involves mitochondrial cytochrome *c* release and subsequent activation of caspase 9, and *via* the death-receptor mediated (extrinsic) pathway, which involves the activation of caspase 8 [41]. Proapoptotic NSAID-induced activation of caspases, mediated by the early release of mitochondrial cytochrome *c* in mammalian cancer cells, has clearly been demonstrated by studies such as that of Piqué et al. [38]. The authors reported that cytochrome *c* release preceded both the disruption of the mitochondrial membrane potential (ΔΨ*m*) and the activation of caspases. The latter observation was confirmed by Zimmermann and coworkers [39] who in turn showed that, in aspirin-induced apoptosis of human cancer cells, the release of mitochondrial cytochrome *c* was itself triggered by translocation of proapoptotic Bax protein to the mitochondria. Conversely, overexpression of antiapoptotic Bcl-2 protein inhibited both cytochrome *c* release and apoptosis of aspirin-treated cancer cells. Furthermore, deletion of apoptotic protease-activating factor-1 (APAF-1), a cytosolic molecule which mediates caspase 9 activation after binding to mitochondrial cytochrome *c*, rendered cells resistant to aspirin-induced apoptosis. These observations indicate that cytochrome *c* release is a critical mediatory mechanism of apoptotic cell death induced by NSAIDs [39]. In fact, it has also been shown that aspirin-induced apoptosis of *S. cerevisiae* cells deficient in manganese superoxide dismutase (MnSOD) and cultivated in nonfermentable ethanol medium is preceded by the early release of cytochrome *c*, followed by a drastic fall in ΔΨ*m* [93]. 

Extrinsic activation of caspase 8 is also an important mediator of NSAID-induced cancer cell apoptosis [42, 48]. Activated caspase 8 can initiate apoptosis by activating downstream caspases or by cleaving the BH3-domain only protein Bid. Truncated Bid (tBid) can translocate to the mitochondria to trigger cytochrome *c* release [94, 95] and can also activate Bax [96]. Indeed, aspirin-induced apoptosis of AGS gastric cancer cells was marked by activation of caspase 8 and Bid cleavage, along with the mitochondrial translocation of Bax, activation of downstream caspases, and cleavage of PARP. Specific inhibition of caspase 8 abrogated cleavage of both Bid and PARP and prevented aspirin-induced AGS cell apoptosis, thus implicating extrinsic caspase activation in the initiation of aspirin-induced apoptosis [42]. However, in this same study, the release of cytochrome *c* and activation of caspase 9 were also observed, thus suggesting the involvement even of mitochondrial-mediated caspase activation. In fact several other studies have since shown that the chemopreventive apoptotic effect of aspirin [41, 43, 45, 46] and modified NSAIDs such as NO-aspirin [97] and phosphosulindac [20] on mammalian cancer cells may involve the initiation of both intrinsic and extrinsic caspase activation pathways, which operate in parallel to one another. 

The ability of NSAIDs to activate caspases largely explains the profound influence they have on Bcl-2 proteins such as Bax, Bid, and Bcl-2 [42, 43], the expression and cellular distribution of which can be greatly altered by NSAIDs to mediate apoptosis in cancer cells. NSAIDs such as aspirin and indomethacin have long been shown to induce cancer cell apoptosis by upregulating the expression of proapoptotic Bcl-2 proteins, such as Bax and Bak, and by downregulating expression of anti-apoptotic Bcl-2 proteins such as Bcl-2 and Bcl-X_L_ [29, 44]. More recent work has shown that NSAID-induced downregulation of Bcl-X_L_ can be induced in part by proteasome-mediated protein degradation [98]. 

NSAID-induced intrinsic and extrinsic activation of caspases, along with modulation of Bcl-2 protein expression, all seem to converge at the mitochondria. Here, these pathways induce events such as the release of cytochrome *c* and other proapoptotic molecules, including SMAC/Diablo, from the mitochondria [43], which irreversibly commit cells to the full apoptotic phenotype. Thus mitochondria clearly constitute a very important target of NSAID-induced apoptosis, as indicated by the many studies associating NSAID-induced apoptosis of mammalian cells with mitochondrial dysfunction, such as uncoupling of oxidative phosphorylation [99], induced mitochondrial permeability transition [100, 101], and inactivation of mitochondrial enzymes such as aconitase and respiratory chain proteins [45]. 

Likewise, it has been shown that the mitochondria of *S. cerevisiae* cells constitute a critical target of NSAIDs such as aspirin [32], the proapoptotic effects of which were shown to be associated with inhibition of the electron transport chain [93]. Similarly, Van Leeuwen and coworkers [33] observed that growth inhibition and apoptosis of *S. cerevisiae* cells caused by treatment with the NSAID diclofenac were due to mitochondrial dysfunctional events involving the inhibition of the electron transport chain. The fact that yeast cells, which are primitive eukaryotes, share a common mitochondrial target of NSAIDs with mammalian cells is highly significant, because it suggests that mitochondria constitute a unifying, dominant target of NSAID-induced apoptosis in all mammalian cancer cell types. This may certainly help inform the design of more effective NSAIDs for chemopreventive purposes and illustrates the important contribution of yeast cells as a complementary experimental model for the study of NSAID-induced apoptotic mechanisms.

### 3.2. Depletion of Polyamines

Polyamines such as spermine, spermidine, and putrescine are abundant polycations found in all eukaryotic cells and play an essential role in cellular development and proliferation [102]. High polyamine levels are in fact associated with the induction of cell proliferation [103, 104], whilst lowered polyamine levels have been found to promote cell growth inhibition [105] and apoptosis [106]. Hence, it is no surprise that the polyamine content of cancer cells tends to be significantly higher than that of normal cells [102], thus representing a potential target of anti-neoplastic agents such as NSAIDs, a number of which have been shown to mediate their chemopreventive effect by promoting the catabolic degradation of polyamines in cancer cells [35, 49, 107]. This takes place by virtue of the general ability of NSAIDs to modulate cellular polyamine metabolism, which is regulated by the biosynthetic enzyme ornithine decarboxylase (ODC) and the catabolic polyamine-acetylating enzyme spermidine/spermine *N* ^1^-acetyltransferase (SSAT) [49, 107]. For instance, indomethacin-induced growth inhibition of human colon cancer cells has been shown to be associated with downregulation of ODC activity and upregulation of SSAT activity, which concurrently impair the synthesis of polyamines and increase the rate at which they are degraded. The consequent depletion of cellular polyamines was accompanied by apoptotic cell death [35]. Other traditional NSAIDs such as aspirin [108], sulindac sulfone [49], and ibuprofen [107] mainly induce the enhanced degradation and export of polyamines by upregulating gene expression of SSAT in cancer cells, resulting in reduced proliferation and increased apoptosis. This is also true of modified NSAIDs such as phosphosulindac [20], the antiproliferative proapoptotic effect of which can, like its NSAID precursor sulindac [109], be enhanced even further by concurrent treatment of cells with ODC inhibitors such as difluoromethylornithine (DFMO) [20, 110, 111]. Both DFMO and phosphosulindac act synergistically to enhance the depletion of polyamines in colon cancer cells, strongly inhibiting their proliferation and greatly enhancing apoptosis [20]. 

### 3.3. Modulation of NF-*κ*B Activity

Nuclear factor kappa B (NF-*κ*B) is a ubiquitous cellular transcription factor which regulates the expression of genes associated with inflammation, immune responses, cell growth, differentiation, and apoptosis. Composed of p65 (RelA) and p50 polypeptides, this complex transcription factor is sequestered in an inactive, heterodimeric form within the cell cytoplasm by I kappa B alpha (I*κ*B*α*) or beta (I*κ*B**β**) inhibitor proteins [112, 113]. Stimulation by appropriate signals (such as proinflammatory cytokines including tumour necrosis factor (TNF)) triggers the IK*α* or IK**β** kinase- (IKK-) mediated phosphorylation of I*κ*B proteins, which consequently undergo ubiquitin-dependent proteasomal degradation. This permits translocation of NF-*κ*B molecules to the nucleus, where they then bind to and promote the transcription of numerous target genes bearing a *κ*B-binding motif [79, 113, 114]. 

The constitutive activation of NF-*κ*B is a hallmark of several types of cancers [115–117] and is heavily associated with cancer cell resistance to cytotoxic agents, due in part to its induced upregulation of anti-apoptotic proteins [118]. Thus, NF-*κ*B constitutes yet another potential target of chemotherapeutic agents such as NSAIDs, which can modulate NF-*κ*B signalling in cancer cells to promote the onset of apoptosis [36, 54, 60, 107, 111, 114, 119].

Traditional NSAIDs such as aspirin have been reported to inhibit NF-*κ*B activation by preventing the degradation of I*κ*B [119]. Aspirin can inhibit TNF-induced I*κ*B*α* degradation [120] by modulation of p38 mitogen-activated protein (MAP) kinase pathways [121] and by disrupting the ubiquitin-dependent proteasomal pathway, of which I*κ*B*α* is a substrate [47]. Aspirin can also block I*κ*B**β** degradation through competitive inhibition of IKK**β**-ATP molecular binding, thus facilitating selective inhibition of IKK kinase (IKK**β**) [114]. All these mechanisms prevent NF-*κ*B activation and subsequent transcription of anti-apoptotic proteins. It has also been shown that the NSAID sulindac specifically inhibits IKK**β** activity and NF-*κ*B activation in cancer cells, thus promoting apoptosis [122]. Likewise, the growth inhibitory effect of NSAIDs such as ibuprofen [51], indomethacin, and etoricoxib, a recently developed COX-2 inhibitor [117], is associated with their inhibition of NF-*κ*B signalling in cancer cells.

The growth inhibitory effect mediated by modified NSAIDs such as NO-NSAIDs, on various cancer cell lines, also involves the modulation of NF-*κ*B signalling [54, 55]. The growth inhibitory effect of NO-aspirin, associated with its ability to reduce proliferation and enhance apoptosis of cancer cells, was shown to be significantly correlated to its profound inhibition of the NF-*κ*B signalling pathway, the occurrence of which was suggested to be due to inhibition of NF-*κ*B binding to DNA in the nucleus [55]. Sun and Rigas [56] went on to demonstrate that proapoptotic inhibition of NF-*κ*B signalling in human colon cancer cell lines, treated with NO-aspirin, stemmed from the latter's induced generation of reactive oxygen and nitrogen species (RONS), which may have interacted directly or indirectly (*via* the redox-sensitive thioredoxin (TRX) system) with NF-*κ*B, impairing its ability to bind to recognition DNA sequences in the nucleus. This is highly conceivable given that NF-*κ*B transcriptional activity is sensitive to redox changes [123]. In fact, it has since been shown that structurally diverse NO-NSAIDs such as NO-aspirin and NO-naproxen can suppress NF-*κ*B signalling in cells *via* S-nitrosylation of the NF-*κ*B transcription factor. This redox-signalling mechanism is mediated by the released NO group which, on binding to the p65 monomer of NF-*κ*B, impairs the transcription factor's ability to bind to DNA [57]. The redox-induced inhibition of NF-*κ*B signalling is thought also to partly mediate the growth inhibitory effect (including apoptosis, cell cycle arrest, and inhibition of proliferation) of phospho-NSAIDs [19, 21, 111] and HS-NSAIDs in cancer cells [60]. 

Intriguingly, the effect of NSAIDs on NF-*κ*B activity seems to be cell-type specific, since aspirin-induced apoptosis of HCT 116 colon cancer cells was shown to be mediated by the activation of NF-*κ*B signalling, rather than its inhibition [36]. Additionally, Din and coworkers [124] observed that aspirin-induced I*κ*B*α* degradation, activation of NF-*κ*B signalling, and apoptosis took place in colorectal cancer cells but not in other malignant cell types. Loss of cellular I*κ*B*α*, which is indicative of NF-*κ*B activation, has also been reported in aspirin-induced apoptosis of both immortalised human endothelial cells [125] and animal models of human colorectal cancer [126]. Likewise, Babbar and coworkers [107] observed that aspirin caused the activation of NF-*κ*B signalling in Caco-2 colon cancer cells, suggesting even that this event was responsible for the upregulation of SSAT expression and polyamine depletion which led to apoptosis. Besides aspirin, other traditional NSAIDs such as diclofenac have also been reported to induce activation of NF-*κ*B signalling as a means to attenuate cancer cell proliferation and promote apoptosis [127]. It has been argued that the varying effects of NF-*κ*B may be due to the specificity by which this transcription factor binds to DNA and activates target genes. Such specificity is in turn dependent on the dimeric composition of the NF-*κ*B complex and on the transcriptional cofactors that it has recruited, both of which can vary depending on the kinetics of induction [128]. Therefore, different stimuli or even the same stimulus exerted under different conditions can induce different NF-*κ*B complexes and different downstream responses [36, 107].

### 3.4. Modulation of MAP-Kinase Activity

Mitogen activated protein (MAP) kinases are serine/threonine-specific proteins which respond to extracellular stimuli and regulate various cellular pathways including mitosis, cell proliferation, survival and death [129]. The three principal MAP kinase subgroups include the extracellular signal-regulated kinases ERK1/2 (p42/p44), c-Jun N-terminal kinases/stress-activated protein kinases (JNKs/SAPKs), and the p38 MAP kinases [121]. NSAIDs such as aspirin and its metabolite salicylate have long been shown to modulate MAP kinase signalling in mammalian cells [130, 131]. This is exemplified by the salicylate-induced activation of p38 MAP kinase signalling in FS-4 fibroblasts, which induced apoptosis [130] *via* a pathway involving the NSAID-induced inhibition of I*κ*B*α* degradation and NF-*κ*B signalling [121]. Based on these observations, the authors concluded that apoptotic cell death induced by p38 MAP kinase activation played an important role in mediating the anti-neoplastic effects of NSAIDs. The important mediatory role of MAP kinase modulation in the context of the anti-neoplastic effects of NSAIDs was further highlighted by Jones and coworkers [132], who demonstrated that NSAID-induced inhibition of angiogenesis involved the disruption of ERK1/2 kinase signalling, a typically prosurvival pathway [133]. Additionally, inhibition of ERK1/2 signalling was shown to be the mechanism by which aspirin sensitises human cervical cancer cells to apoptosis induced by tumour necrosis factor-related apoptosis-inducing ligand (TRAIL) [40]. Modulation of MAP kinases has also been implicated in the suppressive effect of certain NSAIDs such as aspirin upon the factor activator protein (AP-1), a downstream target of MAP kinases which is critical for inducing neoplasia and activation of genes associated with inflammation and infection [134]. 

The modulation of MAP kinases has been shown to be critical for the growth inhibitory effect of modified NSAIDs such as NO-aspirin [135]. Aside from its propensity to inhibit NF-*κ*B signalling, NO-aspirin was shown to induce the activation (marked by increased phosphorylation) of all three main MAP kinases in a concentration-dependent manner, in colon cancer cells. This was caused by the NO-aspirin-induced generation of RONS and subsequent oxidation of cellular thioredoxin-1 protein (Trx1p), which facilitated the proapoptotic autophosphorylation and activation of ASK1, a protein involved in the MAP kinase cascade and only kept inactive when attached to reduced Trx1p [56]. The same authors implicated ASK1-Trx1p cleavage in the activation of MAP kinase signalling, which in turn partly mediated the growth inhibitory effect of NO-aspirin, an effect marked by increased apoptosis and inhibition of cell proliferation. Likewise, NO-aspirin-induced cell cycle arrest and apoptosis of pancreatic cancer cells has been shown to occur *via* ROS-mediated modulation of all three MAP kinase signalling pathways and their downstream effector molecules such as p21 and cyclin D1 [53]. The rapid response of MAP kinases to the presence of RONS is not surprising given their well-established redox sensitivity [136]. The modulation of MAP kinase pathways was also implicated in the chemopreventive effect of NO-sulindac on UVB-induced melanoma cells [59] and in phospho-NSAID-mediated, redox-dependent apoptosis of colon cancer cells [19, 111].

### 3.5. Inhibition of Wnt/*β*-Catenin Signalling

 The Wnt signalling pathway regulates the biosynthesis of **β**-catenin, a protein which is required for cell-to-cell adhesion and involved in the expression of genes associated with cancer [137]. Constitutive activation of Wnt/**β**-catenin signalling has been implicated in the development of numerous human malignancies [138–143]. Aberrant Wnt/**β**-catenin signalling is associated with the nuclear accumulation of **β**-catenin and the subsequent activation of the transcription factor known as T-cell factor (TCF). The resulting **β**-catenin/TCF complex promotes the transcriptional activation of target genes associated with proliferation, such as *cyclin D1 *[144, 145], hence the implicated role of Wnt/**β**-catenin signalling in human carcinogenesis.

The NSAIDs aspirin and indomethacin have been shown to attenuate Wnt/**β**-catenin signalling in colorectal cancer cells, in a dose-dependent manner, by inhibiting the transcription of **β**-catenin/TCF-responsive genes. This NSAID-induced inhibition did not involve cleavage of the **β**-catenin/TCF complex but rather the hyperphosphorylation and consequent stabilization of **β**-catenin, presumably caused by the inactivation of a phosphatase enzyme [146, 147]. Further studies have since shown that aspirin-mediated downregulation of Wnt/**β**-catenin/TCF signalling can indeed be mediated by its induced inactivation of protein phosphatase 2A [148] but also, in part, by the downregulation of upstream specificity protein (Sp) transcription factors [149]. Other NSAIDs, such as sulindac, can also mediate the antiproliferative degradation of **β**-catenin in cancer cells partly by proteasomal degradation and partly by caspase-mediated cleavage [150], whilst others such as diclofenac have been shown to suppress Wnt/**β**-catenin/TCF signalling *via* the activation of NF-*κ*B [127].

The downregulation of Wnt/**β**-catenin/TCF signalling induced by NSAIDs has been associated with the profound growth inhibition of various cancer cell types, in a manner that seems in large part to be due to inhibition of cell proliferation rather than by direct induction of apoptosis [127, 142, 146, 150, 151]. However, the proapoptotic effect of NSAIDs such as sulindac [152] in colorectal cancer cell lines has been shown to involve downregulation of Wnt/**β**-catenin/TCF signalling. Furthermore, Wnt/**β**-catenin signalling was observed to play a key role in directly mediating the proapoptotic effect of aspirin in human mesenchymal stem cells [153]. Regardless, the high prevalence of **β**-catenin downregulation reported in studies of NSAID-induced growth inhibition of cancer cells underlines the importance of this pathway as a chemopreventive target of NSAIDs. 

This is certainly true for modified NSAIDs such as NO-aspirin, the growth inhibitory effect of which is strongly associated with a number of induced cellular events including the profound concentration-dependent inhibition of **β**-catenin signalling in colon cancer cells [54, 58, 145, 154]. In this regard, NO-aspirin is far more effective than aspirin in that, at concentrations far below those required for the inhibition of cell proliferation, it actually prevented formation of the **β**-catenin/TCF complex, whereas aspirin did not [145]. Moreover, at higher concentrations, NO-aspirin can induce caspase-3-mediated cleavage of **β**-catenin itself, leading to a significant decline of cellular**β**-catenin levels and loss of cell-to-cell adhesion amongst colon cancer cells [58]. The significant downregulation of Wnt/**β**-catenin signalling, mediated at least in part by caspase-mediated**β**-catenin degradation, has also been implicated in the growth inhibitory effect of NO-aspirin on leukaemia [97] and breast cancer cell lines [155]. The same applies for phospho-NSAIDs such as phosphosulindac, the growth inhibitory effect of which was shown to involve **β**-catenin degradation in breast cancer stem cells [156]. 

### 3.6. Oxidative Stress and Disruption of Redox Balance

NSAIDs can mediate apoptosis in both malignant cell lines and budding yeast cells by upregulating the generation of ROS and by inducing oxidative stress. Indeed, it has been argued that ROS generation may constitute a central unifying mechanism by which the anti-neoplastic effects of NSAIDs are mediated, given that oxidative stress is coupled with many proapoptotic signals such as NF-*κ*B inhibition and MAP kinase activation [157, 158]. The fact that ROS accumulation also mediates NSAID-induced apoptosis in primitive eukaryotes such as yeast [33] further corroborates this argument. However, the cellular prooxidant behaviour of NSAIDs has often been the subject of controversy due to conflicting reports that NSAIDs such as indomethacin and sulindac [159, 160] scavenge ROS and exert a cytoprotective antioxidant effect in cells. Likewise, there are numerous reports derived from *in vivo* studies of rats showing that NSAIDs such as aspirin can exert a cytoprotective antioxidant effect associated with the attenuation of ROS [161, 162] and of lipid peroxidation [163, 164], along with the upregulation of antioxidants such as reduced glutathione (GSH) [165] and superoxide dismutases (SODs) [166]. It has also been shown that low doses of aspirin can confer long-term cytoprotective resistance against H_2_O_2_-induced oxidative stress in *S. cerevisiae* cells [167].

Nevertheless, other investigations have clearly shown that NSAIDs can induce the proapoptotic accumulation of ROS in both yeast [33] and mammalian cells [157]. For instance, apoptotic cell death of respiring *S. cerevisiae* cells cultivated in the presence of diclofenac was clearly linked to a significant increase in cellular ROS, as measured by the ROS-sensitive fluorescent probe 2′,7′-dichlorodihydrofluorescein diacetate (DCDHF-DA) [33]. Similarly, indomethacin-induced apoptosis of gastric epithelial cells, which was abrogated after treatment with antioxidants such as N-acetylcysteine (NAC), was shown to require the generation of ROS [168], an event likewise implicated in the proapoptotic depletion of polyamines induced by indomethacin in colorectal cancer cells [35]. Additionally, Chan and coworkers [7] showed that human colorectal cancer cell apoptosis induced by both indomethacin and sulindac was marked by the accumulation of arachidonic acid, an event which is itself heavily associated with the accumulation of cellular ROS [83, 169]. The accumulation of ROS was also shown to be a critical inducer of mitochondrial cytochrome *c* release, disruption of  ΔΨ_*m*_, caspase activation, and apoptosis in salicylate-treated mammalian tumour cells [170]. Additionally, the NSAID sulindac and its metabolites have been shown to enhance the antitumour effect of the proteasome-inhibitor bortezomib, primarily through the synergistic generation of ROS [171]. It has been suggested that conflicting reports of NSAID redox behaviour in eukaryotic cells might simply be due to differences in the timing of measurements of ROS and antioxidant changes in experimental setups, given that cellular antioxidant levels are naturally expected to increase in subsequent response to elevated ROS [157]. 

The molecular mechanisms underlying NSAID-induced proapoptotic generation of ROS in mammalian and yeast cells have not yet been fully elucidated. However, it is well known that mitochondria, a major source of cellular ROS [172] and a central component of the apoptotic machinery, are profoundly affected by NSAIDs in both yeast [33, 93] and mammalian cells [99, 101]. For example, the aspirin metabolite salicylate has been shown to inhibit the mitochondrial electron transport chain in mammalian cells by interacting with an Fe-S cluster of Complex I, through its o-hydroxyl group. This was found to induce ROS accumulation and oxidative stress, which in turn caused proapoptotic events such as mitochondrial permeability transition and cytochrome *c* release [173]. Likewise, aspirin-induced cell cycle arrest and apoptosis of HepG2 hepatoma cells were shown to be induced by ROS accumulation and increased oxidative stress, accompanied by severe mitochondrial dysfunction such as the inactivation of electron transport chain proteins and aconitase [45]. Van Leeuwen et al. [33] observed that reduced cell growth and viability of *S. cerevisiae* yeast cells treated with diclofenac are due to mitochondrial dysfunction associated with the inhibition of electron transport chain subunit proteins Rip1p (of Complex III) and Cox9p (of Complex IV). This caused inhibition of cell respiration and subsequent ROS production, resulting in cell death. Inhibition of cellular respiration induced by aspirin was also observed in MnSOD-deficient yeast cells cultivated in ethanol medium [93], and recent studies in our laboratory have established the aspirin-induced proapoptotic generation of mitochondrial superoxide radicals in these cells (unpublished work). 

It has been shown that the proapoptotic induction of oxidative stress induced by NSAIDs such as aspirin is strongly associated with the modulation of cellular redox homeostasis. This is exemplified by the observed increase of aspirin-induced apoptosis in HepG2 cells with GSH depletion and compromised redox balance [174]. In addition to this, studies in *S. cerevisiae* yeast cells have shown that aspirin-treated MnSOD-deficient yeast cells grown in ethanol medium experienced a very significant decrease in cellular reducing power with respect to wildtype cells, as measured by the NADPH/NADP^+^ concentration ratio. This was accompanied by a significant decrease of the GSH/GSSG concentration ratio, owing to a buildup of GSSG, prior to cell death [175]. 

The induction of oxidative stress mediated by disruption of cellular redox balance is also central to the apoptotic effect of modified NSAIDs in cancer cells [53, 56, 58, 158]. For instance, oxidative stress induced by NO-aspirin in colon cancer cells was shown to be mediated by the depletion of cellular GSH, caused by the latter's association with the spacer component of NO-aspirin and subsequent formation of a GSH conjugate. The resulting redox imbalance then initiated a number of downstream proapoptotic pathways such as **β**-catenin cleavage, inhibition of Wnt signaling, and mitochondrial-mediated activation of caspases [58]. Sun and Rigas [56] further demonstrated that redox-induced apoptosis of colorectal cancer cells treated with NO-aspirin involved the generation of RONS, the growth inhibitory effect of which was mediated by oxidative alteration and impairment of the thioredoxin redox system. Oxidised thioredoxin-1 induced MAP kinase activation and NF-*κ*B inhibition, both of which are critical mediatory pathways of NO-aspirin-induced apoptosis [55, 135].

Oxidative stress, marked by RONS accumulation and redox imbalance associated with the suppression of GSH and increased oxidation of Trx-1, also plays a central role in apoptosis induced by phospho-NSAIDs [19–21, 56, 110, 111]. Like NO-NSAIDs, the strong prooxidant effect exerted by phospho-NSAIDs is reported to set in motion a pleiotropic cascade of redox-sensitive signalling events including activation of MAP kinases and inhibition of NF-*κ*B signalling [19, 21, 56] along with the depletion of cellular polyamines, at least in the case of phosphosulindac [20, 110, 111]. The collective initiation of all these antiproliferative, proapoptotic pathways accounts for the very potent growth-inhibitory effects of phospho-NSAIDs, with respect to their traditional NSAID precursors [111]. Likewise, recent studies have shown that both apoptosis and cell cycle arrest of cancer cells treated with HS-NSAIDs, such as HS-aspirin, are induced as a result of oxidative stress and redox imbalance [60]. 

### 3.7. Other NSAID-Induced Proapoptotic Pathways

Further mechanisms by which NSAIDs can promote apoptosis in malignant cells include the induced depletion of survivin, an inhibitor of apoptosis protein which regulates the cell cycle and apoptosis in eukaryotic cells. Survivin expression in cancers tends to be very high and is in fact associated with tumour cell chemoresistance, making it an attractive target of antineoplastic treatments [176], including NSAIDs. Lu and coworkers [177] showed that aspirin caused significant and targeted depletion of survivin in breast cancer cells by upregulating its proteasomal degradation, consequently sensitizing the tumour cells to TRAIL-induced apoptosis. Moreover, aspirin acted synergistically with TRAIL to promote apoptosis of the breast tumour cells. Similarly it has been shown that cell cycle arrest and apoptotic cell death induced by the NSAIDs ibuprofen [51] and tolfenamic acid [52] in human colon and prostate cancer cells, respectively, are accompanied by significant depletion of survivin levels.

Another COX-independent proapoptotic pathway induced by NSAIDs is the impairment of proteasome function, as demonstrated by Dikshit and co-workers [47], who observed a time- and dose-dependent decline of proteasomal activity in neuroblastoma cells treated with aspirin. Accompanied by the accumulation of ubiquitylated proteins and profound mitochondrial abnormalities, the aspirin-induced impairment of proteasomal function was shown to activate the intrinsic apoptotic pathway, marked by a release of cytochrome *c* and the activation of caspase 9. 

Finally, NSAIDs such as sulindac have been reported to induce sensitization of cancer cells to *mda7/IL24*-mediated apoptosis. The *mda7* gene, also known as *IL24*, is of the interleukin (IL) 10 family of cytokines [178, 179]. Ectopic expression of *mda7* is known to exert a potent tumour-suppressive effect against a variety of human cancer cells, with little to no effect on normal cells [180–182]. Furthermore, intratumoural administration of adenoviral vectors which express *mda7* (Ad-mda7) has been shown to exhibit antitumour and antiangiogenic activity in human lung tumour xenografts [183]. Also, Oida and coworkers [50] demonstrated that Ad-mda7-mediated growth inhibition and apoptosis of human lung cancer cells was greatly enhanced by concurrent administration of sulindac, which increased the half-life of MDA7 protein in the cells. This resulted in the increased expression of MDA7 protein and of its proapoptotic downstream effector proteins including p38MAPK, caspase-9, and caspase-3, consequently sensitizing the lung cancer cells to apoptosis. Therefore, sulindac essentially altered MDA7 protein turnover in lung cancer cells in such a way as to promote apoptotic cell death [50].

## 4. Concluding Remarks

**Figure 2:**
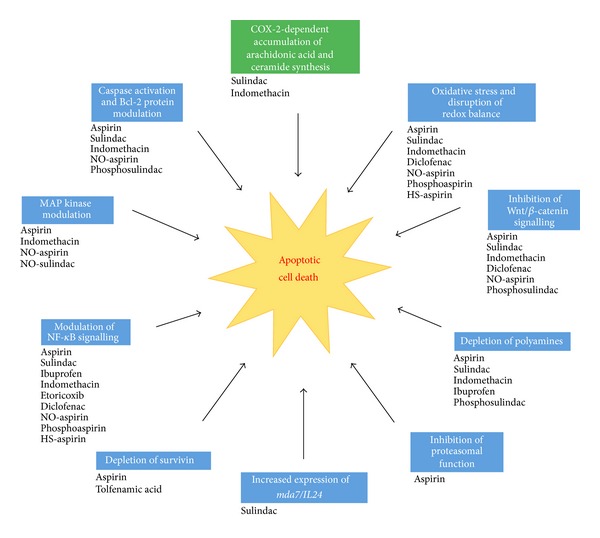
The major proapoptotic pathways induced by NSAIDs. Both traditional and modified NSAIDs have been shown to induce apoptosis in eukaryotic cells by initiating mechanisms which are largely independent of COX inhibition (shown in blue), with the exception being the COX-2-dependent accumulation of arachidonic acid and subsequent synthesis of ceramide induced by sulindac and indomethacin (shown in green). Important COX-independent proapoptotic pathways induced by NSAIDs include caspase activation and modulation of Bcl-2 proteins, depletion of polyamines, modulation of NF-*κ*B signalling and of MAP kinase activity, inhibition of Wnt/*β*-catenin signalling, inhibition of proteasomal function, depletion of survivin, increased expression of *mda7/IL24* and also oxidative stress associated with mitochondrial dysfunction, ROS accumulation and the disruption of cellular redox balance.

Ongoing investigations of proapoptotic mechanisms underlying the promising anti-neoplastic properties of NSAIDs such as aspirin remain a top research priority, since an improved understanding of such pathways will help to enhance current anticancer drug treatments [2]. What is certain thus far is that NSAIDs generally exert a dose-dependent pleiotropic effect on cancer cells (see Figure 2), initiating a very complex cascade of signalling events which collectively induce apoptosis. Although much more remains to be elucidated, there is also mounting evidence which suggests that NSAID-induced signalling events associated with the induction of oxidative stress, such as mitochondrial dysfunction and altered redox signalling, may be the dominant pathways underlying all other proapoptotic effects induced by NSAIDs in cancer cells [184]. 

Consistent with the growing number of mammalian cell studies implicating oxidative stress as the dominant pathway of NSAID-induced growth inhibition [185], the proapoptotic effects of NSAIDs in yeast cells are likewise associated mainly with mitochondrial dysfunction, ROS generation, and redox imbalance. In particular, yeast cell studies have highlighted the pivotal importance of mitochondrial MnSOD as a cytoprotective defence against NSAIDs such as aspirin [32]. This implies that specific, targeted modulation of mitochondrial MnSOD can be exploited to enhance NSAID-induced oxidative stress and apoptosis in malignant mammalian cells.

It is currently hypothesised that cancer cells constantly experience much higher levels of oxidative stress with respect to normal cells, due to their increased metabolic rate [186]. Because of this, cancer cells are more reliant on antioxidant enzymes such as MnSOD and are thus believed to be far more sensitive to perturbations in redox balance compared to normal cells [16]. In fact, it has been shown that silencing of MnSOD, using anti-sense MnSOD antibodies, amplifies ROS accumulation and apoptosis in squamous cell carcinomas exposed to gamma radiation and anticancer drugs [187]. Moreover, it has recently been shown that silencing of MnSOD messenger RNA, using small interfering RNA (siRNA), amplified apoptosis of melanoma cells induced by the NSAID diclofenac [16]. This same study also demonstrated that diclofenac-induced accumulation of ROS, depletion of MnSOD expression and activity, and apoptosis were specific to melanoma cells.

In light of all these lines of evidence compiled through complementary study of both mammalian and yeast cell models, the induction of oxidative stress and redox imbalance induced by NSAIDs, together with the targeted modulation of mitochondrial MnSOD, merits serious consideration for future investigations. 

## Figures and Tables

**Table 1:** Human cancer cell targets of the proapoptotic effects of prominent traditional and modified NSAIDs.

NSAID type	Target cell type [references]
*Traditional NSAIDs *	
Aspirin	Colon cancer cells [34–36], leukaemia cells [37, 38], cervical cancer cells [39, 40], gastric cancer cells [41–44], hepatocellular carcinoma cells [45, 46], endometrial cancer cells [29], neuroblastoma cells [47]
Indomethacin	Colon cancer cells [7, 35], Gastric cancer cells [44], Endometrial cancer cells [29]
Sulindac	Colon cancer cells [6, 7, 48, 49], prostate cancer cells [48], hepatocellular carcinoma cells [8], lung cancer cells [50]
Ibuprofen	Colon cancer cells [51]
Diclofenac	Neuroblastoma cells [15], melanoma cells [16]
Tolfenamic Acid	Prostate cancer cells [52]
*Modified NSAIDs *	
NO-Aspirin	Pancreatic cancer cells [53], colon cancer cells [17, 54–58]
NO-sulindac	Colon cancer cells [17], melanoma cells [59]
NO-ibuprofen	Colon cancer cells [17]
NOSH-aspirin	Colon cancer cells [24]
Phosphoaspirin	Colon [19, 21, 56], pancreatic [21], and breast [21] cancer cells
Phosphosulindac	Colon, pancreatic, and breast cancer cells [21]
HS-aspirin	Colon cancer cells [22], breast cancer cells [60]
HS-ibuprofen	Colon cancer cells [22]
HS-naproxen	Colon cancer cells [22]
HS-sulindac	Colon cancer cells [22]
